# Expression profiling of human glial precursors

**DOI:** 10.1186/1471-213X-8-102

**Published:** 2008-10-23

**Authors:** James T Campanelli, Robert W Sandrock, Will Wheatley, Haipeng Xue, Jianhua Zheng, Feng Liang, Jonathan D Chesnut, Ming Zhan, Mahendra S Rao, Ying Liu

**Affiliations:** 1 Q Therapeutics, Inc. 615 Arapeen Dr., Ste. 102, Salt Lake City, UT 84108, USA; 2 Invitrogen Corporation, 5781 Van Allen Way, Carlsbad, California 92008, USA; 3 Bioinformatics Unit, Branch of Research Resources, National Institute on Aging, NIH, Baltimore, MD 21224, USA

## Abstract

**Background:**

We have generated gene expression databases for human glial precursors, neuronal precursors, astrocyte precursors and neural stem cells and focused on comparing the profile of glial precursors with that of other populations.

**Results:**

A total of 14 samples were analyzed. Each population, previously distinguished from each other by immunocytochemical analysis of cell surface markers, expressed genes related to their key differentiation pathways. For the glial precursor cell population, we identified 458 genes that were uniquely expressed. Expression of a subset of these individual genes was validated by RT-PCR. We also report genes encoding cell surface markers that may be useful for identification and purification of human glial precursor populations.

**Conclusion:**

We provide gene expression profile for human glial precursors. Our data suggest several signaling pathways that are important for proliferation and differentiation of human glial precursors. Such information may be utilized to further purify glial precursor populations, optimize media formulation, or study the effects of glial differentiation.

## Background

Glial differentiation is a specific developmental process that has been extensively characterized in both rodents and human [1]. Embryonic stem cells generate a neuroectoderm that undergoes rostrocaudal and dorsoventral patterning and significant expansion to make a large number of neural stem cells (NSCs). NSCs first generate neuronal precursors and subsequently differentiate into glial precursors that further mature into two major types of glia: oligodendrocytes and astrocytes. During this process, multiple growth factors, transcription factors and molecules of different signal transduction pathways are involved, including fibroblast growth factors (FGFs), epidermal growth factors (EGFs), transforming growth factors beta (TGFβ) family members, Notch-Hes, inhibitor of DNA-binding (ID) family and Wnt pathways (reviewed in [2,3]). Identification of these molecules (markers) is important for the isolation, purification, and characterization of human glial precursors, which may find extensive applications in transplantation studies and regenerative medicine.

Studies of glial development and differentiation in rodents have identified antibodies that recognize markers for isolating human glial cells [4-7]. For instance, A2B5, which reacts with ganglioside epitope GT3 [8], characterizes a glial precursor population [6,7]. Upon further differentiation, A2B5+ cells give rise to oligodendrocyte precursors that express PDGFRα, Sox10, and NG2 [2,3]. Multiple lineage pathways have been suggested for astrocyte development [9], including maturation of radial glia [10,11], a neuron-astrocyte precursor and an oligodendrocyte-astrocyte precursor [7,12-15]. Our laboratory has used antibodies to an extracellular matrix transmembrane protein CD44 to isolate astrocyte precursors from rat, mouse and human neural tissue. These CD44+ cells only gave rise to astrocytes in vitro and in vivo and did not differentiate into neuronal or oligodendrocytic lineages, even in conditions where glial progenitors or stem cells readily differentiated into such phenotypes [13]. Growth factors that are important for glial differentiation include bone morphogenetic proteins (BMP) 2 and 4, leukemia inhibitory factor (LIF), and ciliary neurotrophic factor (CNTF, [16-18]).

Although there are significant similarities in the differentiation of glia in chick, rodent and human systems, there are nevertheless differences as well. For example, A2B5, which characteristically labels glial precursors in rodent cells, has been reported to occasionally recognize cells of the neuronal lineage derived from human embryonic stem cells (hESCs) [5]. This is not surprising since even between rat and mouse spinal cord, the A2B5 staining pattern is slightly different (Liu and Rao, unpublished). Likewise the pattern of CD44 expression in mouse and rat is distinct [19,20] and the growth factor requirements of early precursors appears to differ as well [21].

We have previously created large scale gene expression profiles of hESCs, NSCs and multipotent neural precursor cells and identified a list of markers, which can be used to generate antibodies, design PCR primers, and characterize developmental stages for human neural cells [4,22]. In this manuscript, we extended these studies to compare human glial progenitors to NSCs and more differentiated progeny. We confirmed the utility of some of the glial precursor markers that were defined in rodent, and proposed novel markers and signaling pathways that may be important for proliferation and differentiation of human glial precursor cells.

## Methods

### Preparation of fetal brain derived cell populations

Tissue from fetal cadavers of gestational age 20 to 23 weeks was procured by Procurement Specialists employed by Advanced Bioscience Resources (ABR) following Donor ID and Informed Consent SOPs, Donor Medical Record Review procedures. All protocols and procedures were reviewed by the Western Institutional Review Board and deemed that any further IRB oversight was unnecessary. Each population of cells included in the present study was derived from a different biological donor.

**Figure 1:**
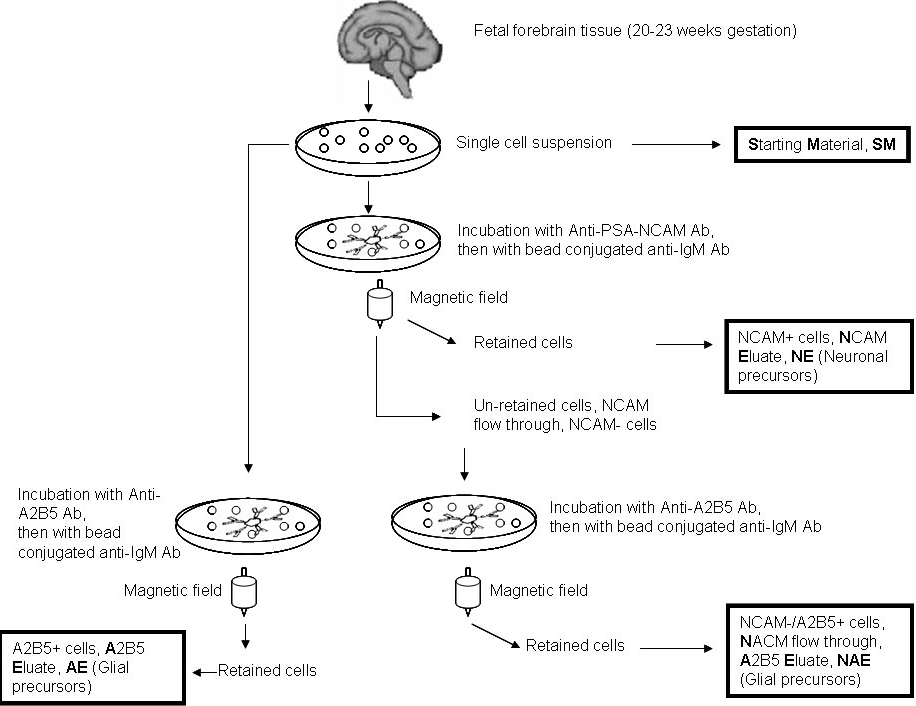
Flow chart of generation of selected samples using MACS or double MACS in this work.

The isolation of the cells used in the present study is described as follows (Figure 1). Fetal forebrain was dissociated into a single cell suspension using enzymatic and mechanical methods according to published procedures and these cells are named as Starting Material: SM [23]. Purified precursors were obtained by single or double immunomagnetic cell sorting (magnetic cell sorting, MACS) using Miltenyi superparamagnetic bead technology according to the manufacturer's instructions. Briefly, the single cell suspension was incubated in anti-PSA-NCAM antibody (NCAM; 1:5000, Millipore, MAB5324) followed by incubation with bead conjugated anti-IgM antibody (Mitenyi). The NCAM positive cells were purified from the mixture first by passage through a magnetic field, these cells were collected and termed NE for NCAM Eluate. Un-retained cells (NCAM negative, i.e., NCAM flow through) were collected afterwards and incubated with A2B5 antibody (1:1000, R&D Systems, MAB1416) followed by incubation with bead conjugated anti-IgM antibody before they were passaged through a second magnetic field. Then the retained cells were collected. These cells were immunologically NCAM- and A2B5+, and were named NAE, for NCAM flow through, A2B5 Eluate). AE, another type of population used in this study was obtained by incubating single cell suspension with anti-A2B5 antibody followed by bead conjugated anti-IgM antibody. The retained cells were A2B5+ and hence named AE (A2B5 Eluate).

### Cell Culture

Human fetal tissue derived glial restricted precursor cell populations (NAE and AE, as described above) were harvested and grown on poly-L-ornithine and laminin-coated dishes for six days in vitro before RNA was extracted for analysis. Cells were grown in DMEM/F12 (with L-glutamine and 15 mM Hepes, no phenol red, Invitrogen), 0.01% human serum albumin, 1× N1 supplement (Sigma), Pen/Strep Fungizone, basic fibroblast growth factor (20 ng/ml), platelet derived growth factor AA (10 ng/ml), and Neurotrophin-3 (5 ng/ml). Human astrocyte precursor cells (hAPCs, CD44+) were derived from human neural precursor cells (ScienCell Research Laboratories) and cultured as described [20]. Culture of other cell populations was described in Shin et al [22]. Unpurified cells (SM) and NCAM+ cells (NE) were not grown in culture and RNA was extracted shortly after cell purification.

### Immunocytochemistry

NAE, NE, AE and SM cells were seeded on chamber slides (LabTek) and grown overnight before they were fixed with 2% paraformaldehyde. For cell surface antigens, cells were blocked with detergent free blocking buffer (10% normal goat serum and 5% bovine serum albumin in PBS) and for internal antigens Triton X-100 and 0.05% Tween-20 were added to the blocking buffer. The following antibodies were used: A2B5 (1:1000), PSA-NCAM (1:5000), PDGFRα (BD Pharmingen, 1:200), NG2 (Millipore, 1:200), GFAP (DAKO, 1:4000), Tuj1 (Sigma, 1:4000), Nestin (BD Bioscience, 1:200) and NeuN (Sigma, 1:200). Appropriate fluorescent labeled secondary antibodies (Invitrogen) were used and nuclei were revealed by counter staining with DAPI.

### Illumina bead array, statistical analysis and RT-PCR verification

RNA isolation, cDNA synthesis and Illumina bead array were performed as described [4,24]. Briefly, RNA was isolated from sorted or cultured cells using TRIzol (Invitrogen) and 100 ng total RNA was used for amplification and hybridization to Illumina HumanRef-8 BeadChip according to the Manufacturer's instructions (Illumina, Inc., San Diego, CA; . Array performed by microarray core facility at the Burnham Institute for Medical Research). Array data were processed using Illumina BeadStudio software. Background and rank invariant method [25,26] were used for normalization. Differential gene expression analysis were done using TMEV program in the TM4 software package [27]. Before analyses, all populations that have biological or technical replicates were categorized into six groups, including NAE (A2B5+/NCAM- double sorted cells, two biological replicates), NE (NCAM+ single sorted cells, two biological replicates), AE (A2B5+ single sorted cells, three biological replicates), hAPC (CD44+ cells, three technical replicates), HOPC (A2B5+/NCAM-, fluorescence activated cell sorting, or FACS-purified cells, two technical replicates), and WBFB (WB-015 and FB-017, regarded as two biological replicates of NSCs), and only average values were used for further analysis along with samples that did not have replicates, i.e., SM, hAST line and hSC line. Differentially expressed genes between NAE and NE, AE, WBFB, HOPC and hAPC samples were identified by t-test [28]. Gene expression levels were considered significant only when their detection values were ≥ 0.99 (i.e., p-value ≤ 0.01). Selected genes were verified by RT-PCR using GAPDH as an internal control. Primer sequences in a 5' to 3' format are presented in Table 1.

**Table 1:** Primers for RT-PCR

Gene	Forward primer	Reverse primer
C17	cgacttcaacctcctgcagg	ttctctggtctcagttcccttagc
CHAD	acccagctccacccagcagtg	agaacgtcctggcaggtggg
DACH	aaaatggtggatctgagggg	ttgagtcctcttaggaggcctt
GSTM1	tcaagctatgaggaaaagaagtacacg	tcagggagttcctccaagtactttg
MMP7	tacagtgggaacaggctcaggactat	gccccacatgtttaaagcctttg
IGSF1	agttctagagttggaggcacca	ctgagattcaggctttctccag
LOC150221	attacaaccccctggtgatgtc	taaaccatgaccacgtctcctg
PIP3-E	gaaagagcatctgaatgcaag	tcagcagcagacaaactgttaac
SLC17A8	tgtcaaccacctggacattg	tctggacttcacaattctggg
TNFAIP6	gcctattgctacaacccaca	caaggtcatgacatttcctgtac

## Results

### Human glial precursors and neuronal precursors can be isolated from fetal tissue

We have established transcriptional profiles of 14 cell populations derived or purified from human fetal brain tissues by five different laboratories (Table 2). These include seven glial precursors derived by two methods, FACS and MACS; two neuronal precursors, a population of astrocyte precursors (run in triplicate), an immortalized human astrocyte line, an immortalized Schwann cell line, and two populations of NSCs in the form of neurospheres derived from whole brain and frontal brain. A population of unsorted fetal brain cells (Starting Material, SM) was also included as a control. This SM sample showed similar expression profile to the other seven SM samples examined in a parallel experiment which will be reported in a separate manuscript. In the sample list for the current study, the potential glial precursors NAE (A2B5+/NCAM-), were obtained from fetal brains of 20 weeks and 21 weeks of age by magnetic bead separation using a double sorting method. Cells were first incubated with NCAM antibody, then the NCAM+ population was depleted and NCAM negative cells collected. After subsequent incubation with the A2B5 antibody, A2B5+/NCAM- cells were sorted by magnetic beads. These cells were named NAE (NCAM depleted, A2B5 column Eluate). FACS-purified A2B5+/NCAM- cells (HOPC) [22] were included to serve as a control to the MACS-sorted NAE cell population.

**Table 2:** Samples included in this study

Bar Code	No.	Name	Description	Fetus age	Source
1521406071H	**1**	SM060310	Unsorted fetal brain cells	20 weeks	Q therapeutics
1521406071F	**2**	NAE050421	A2B5+, PSA-NCAM- cells	20 weeks	Q therapeutics
1521406071G	**3**	NAE060615	A2B5+, PSA-NCAM- cells	23 weeks	Q therapeutics
1521406071C	**4**	AE060609–93	A2B5+ cells	21 weeks	Q therapeutics
1521406071D	**5**	AE060617	A2B5+ cells	21 weeks	Q therapeutics
1521406071E	**6**	AE060309	A2B5+ cells	20 weeks	Q therapeutics
1521406071A	**7**	NE060309	PSA-NCAM+ cells	20 weeks	Q therapeutics
1521406071B	**8**	NE060310	PSA-NCAM+ cells	20 weeks	Q therapeutics
1334328027F	**9**	HOPC	A2B5+, PSA-NCAM- cells	NA	Steve Goldman
1334328026C	**10**	HOPC replicate	A2B5+, PSA-NCAM- cells	NA	Steve Goldman
1400364009C	**11**	WB-015	Neurosphere from whole brain	NA	Cognate
1400364009D	**12**	FB-017	Neurosphere from frontal brain	NA	Cognate
1400401019A	**13**	hAPC	CD44+ cells	20–22 weeks	Mahendra Rao
1334328022D	**14**	hAPC replicate	CD44+ cells	20–22 weeks	Mahendra Rao
1334328023C	**15**	hAPC replicate	CD44+ cells	20–22 weeks	Mahendra Rao
1400401019B	**16**	hAST line	Human astrocyte line	NA	Ahmet Hoke
1400401019C	**17**	hSC line	Human Schwann line	NA	Ahmet Hoke

**Figure 2:**
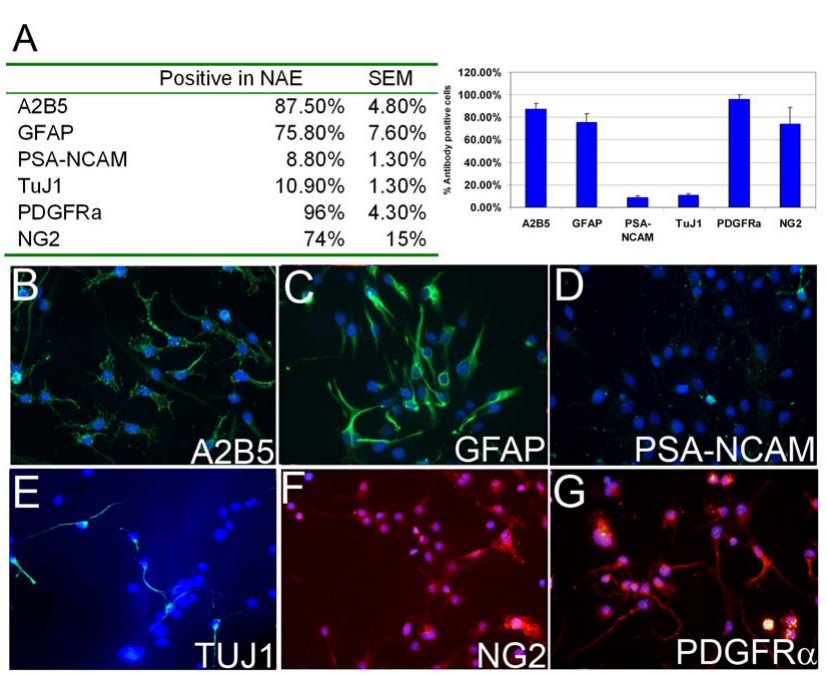
**A2B5+/PSA-NCAM- MACS-sorted cells show properties of glial precursors**. Immunocytochemistry analysis was done for the double MACS-enriched A2B5+/PSA-NCAM- (NAE) cells. Approximately 87% of NAE cells are A2B5+ (B) and ~9% are PSA-NCAM+ (D), They have high immunoreactivity to several glial (GFAP; C) and glial precursor markers (NG2 and PDGFRα; F and G). In contrast, lower percentage of cells show markers specific for the neuronal lineage, such as PSA-NCAM (D) and TuJ1 (βIII tubulin, E).

As an assessment of purity, the glial precursor NAE cells were immunocytochemically characterized. Approximately 88% of the NAE (A2B5+/NCAM-, MACS sorted) cell population stained positive for the glial precursor marker A2B5 (Figure 2B), and ~9% stained positive for the neuronal precursor marker NCAM (Figure 2D). Compared with the unsorted starting material (SM), cells of neuronal lineage such as NeuN+ (not shown) or βIII tubulin (Tuj1)+ cells (Figure 2E) were effectively depleted in the NAE population. NAE cells expressed several known glial markers such as GFAP (76%), NG2 (74%), and PDGFRα (96%, Figure 2C, F, G). These results, combined with the data that were reported for the FACS purified glial precursors (HOPC, A2B5+/NCAM-), NSCs (whole brain and forebrain derived neurospheres) [22], and hAPC (astrocyte precursors, CD44+) [4,20], indicated that distinct populations of precursors could be isolated and purified.

**Figure 3:**
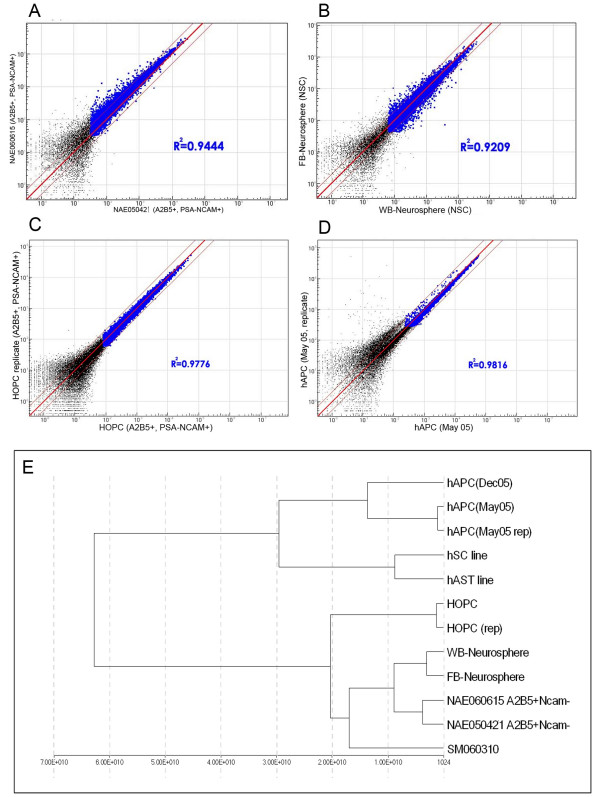
**Scatter plots and dendrogram of related samples.** Biological replicates of MACS sorted glial precursors (NAE) (A), and neurospheres (B), and technical replicates of FACS sorted glial precursors (HOPC) (C), and astrocyte precursors (hAPC) (D) show high R^2 ^values indicating the array data are reliable. The dendrogram generated using BeadStudio software based on Euclidean distance (E) shows that replicates and samples with similar biological properties cluster closer to each other.

We then went on to perform large scale gene expression profiling using Illumina bead array for all 14 samples including technical replicates (Table 2). We chose the Illumina array platform because it has been proven to be reliable, highly sensitive and cost effective [22,24,29]. This platform contained ~24,000 transcripts derived from the Human RefSeq database including full-length and splice variants. Internal reliability for every transcript was achieved by averaging signals obtained from ~30 beads for the same gene or transcript. Before any analysis was performed, the quality of each sample was evaluated by two methods; one means was to assess the quality of the array by analyzing biological or technical replicates in the same run for their general expression patterns; the other means was to examine whether known markers that characterized these populations could be detected using the array system. As shown in Figure 3, biological replicates of glial precursor populations (NAE) and NSCs (neurospheres) derived from either whole brain (WB) or forebrain (FB) were quite similar (R^2 ^= 0.94 and 0.92 respectively; Figure 3A and 3B). Technical replicates for HOPCs and hAPCs also showed significant overlap (R^2 ^= 0.98 for both; Figure 3C and 3D). Two populations of neuronal precursors (NE) did not have a high R^2 ^value, possibly due to the difference in gestational ages or the nature of the single column purification (data not shown). In summary, these results show that replicate samples in the present work cluster closely to each other, which allows for a robust analysis of the similarities and differences between these populations.

Based on the properties of this array platform, only when the detection value is ≥ 0.99 (i.e., detection p value ≤ 0.01), can the corresponding average signal be regarded as "detected" or "expressed". Generally speaking, when detection value is ≥ 0.99, the signal intensity value (i.e., gene expression level) is almost always ≥ 30. In order to further restrict the analysis to improve the reliability of the results, we used a higher cutoff of 100 on signal intensity values. On average, our cell populations expressed 6,200 to 8,900 transcripts at intensity ≥ 100 (Table 3), which was similar to the numbers detected in our earlier reports using Illumina array. Consistent with previous analyses, genes with the highest abundance were ribosomal and structural genes (see Additional file 1).

**Table 3:** Signal abundance of all samples

Samples	Signal ≥ 100	%
SM060310	6221	28.0
NAE050421	6453	29.1
NAE060615	7723	34.8
AE060609–93	7807	35.2
AE060617	7491	33.7
AE060309	6649	30.0
NE060309	7242	32.6
NE060310	6908	31.1
HOPC	8016	36.1
HOPC (replicate)	7978	35.9
WB-015	7911	35.6
FB-017	7892	35.5
hAPC	7794	35.1
hAPC (replicate 1)	8901	40.1
hAPC (replicate 2)	8671	39.1
hAST line	7302	32.9
hSC line	8061	36.3

After implementing a signal intensity value cutoff of ≥ 100, we next examined the expression of several known positive and negative glial markers across these samples. Known glial markers, such as SOX10, PDGFRA, S100B, NKX2.2, OLIG2, EGFR, NESTIN, and OLIG1, had higher expression level in glial precursor NAE than in other populations (Table 4). In contrast, expression of neuronal lineage-related genes such as NPAS1 (neuronal PAS domain protein), DCX (doublecortin), CHRNA3 and CHRNA5 (cholinergic receptor nicotinic alpha polypeptide 3 and 5), ENO2 (enolase 2), and NEF3 (neurofilament 3, 150 kDa), was downregulated compared to the unsorted population SM and the sorted neuronal population NE, supporting the conclusion that NAE was a population enriched for glial precursors but not for neuronal lineage (Table 5).

**Table 4:** Fold change in the expression of selected known glial markers in glial precursors NAE versus neuronal precursors NE and unsorted population SM

Symbol	Synonym	NAE/NE	NAE/SM
SOX10	DOM;WS4;MGC15649	3.4	19.8
PDGFRA	CD140A;PDGFR2	2.9	80.4
S100B	NEF;S100	2.8	10.7
NKX2-2	NKX2B;NKX2.2	2.5	22.0
OLIG2	BHLHB1;RACK17;PRKCBP2	2.4	25.5
EGFR	ERBB;ERBB1	2.3	40.9
NES	FLJ21841	2.0	6.5
OLIG1	BHLHB6	2.0	58.6

**Table 5:** Expression of neuronal specific genes is downregulated in glial precursors NAE versus neuronal precursors NE and unsorted population SM

Symbol	Synonym	NAE/NE	NAE/SM
NPAS1	MOP5	0.09	0.06
DCX	DC;DBCN;LISX;SCLH;XLIS	0.33	0.31
NEF3	NFM;NEFM;NF-M	0.53	0.82
ENO2	NSE	0.62	0.78
CHRNA5		0.67	0.43
NCAM1	CD56;NCAM;MSK39	0.69	1.87
CHRNA3		0.82	0.61

### Glial precursors show distinct gene expression profiles

**Figure 4:**
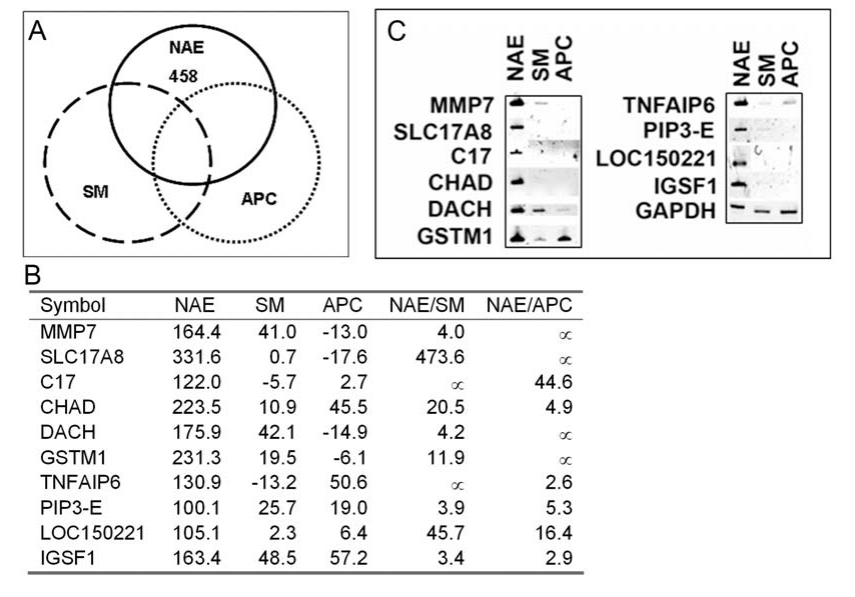
**Unique genes that are expressed in glial precursors NAE.** (A) shows the extent of overlap for expressed genes of three distinct types of cell populations, NAE, unsorted SM and astrocyte precursor hAPC. A total of 458 transcripts are expressed at ≥ 100 as detected by the array uniquely to NAE (detailed gene list can be found in Additional file 2). A selected group of genes (B) are validated by RT-PCR (C). In (B), the numbers in columns NAE, SM and APC represent the expression levels of these samples. The units are arbitrary as the signals are obtained by comparing with the background signals of the array chip. The numbers in columns NAE/SM and NAE/APC represent the ratios of the expression levels of the listed genes. The RT-PCR results (C) validate that all 10 genes listed in (B) have higher expression for NAE than for SM or APC, while the expression for the internal control GAPDH is similar across all three samples.

Similarities and differences of all samples included in the present work were determined, with a focus on obtaining a list of novel glial precursor markers. A Venn diagram using gene expression intensities of NAE (glial precursors), APC (astrocyte precursors) and SM (unsorted starting material) is shown in Figure 4A. A total of 5,929 transcripts were commonly expressed (at an intensity of ≥ 100 in NAE, SM and APC). Four hundred and fifty eight transcripts were uniquely expressed in the glial precursor population NAE. Among these 458 transcripts, we validated 10 by RT-PCR (Figure 4B and 4C), which were among the 344 genes that were expressed more than two fold higher in NAE than in other cell types based on the array data (see Additional file 2).

### Novel cell surface markers can be identified for glial precursors

In search of novel cell surface markers from A2B5+/NCAM- magnetically sorted glial precursor NAE cells, we compared NAE with unsorted fetal brain cells (SM), and with NCAM+ sorted neuronal precursor NE cells. We have found that the expression of several known glial specific cell surface markers, such as NG2 (chondroitin sulfate proteoglycan 4, melanoma-associated, CSPG4), was upregulated in NAE. BCAN, the gene encoding the proteoglycan molecule Brevican,, was found to be enriched in NAE as its expression was nearly 15-fold higher in NAE than in unsorted brain cells SM and 2–4 fold higher than in NCAM+ sorted NE and A2B5+ single sorted AE cells (the signal intensity was 6873.3 in NAE, 466.2 in SM, 2930.8 in NE and 1561.9 in AE). Another group of cell surface molecules that were differentially expressed by NAE were members of the integrin family, particularly integrin beta 5 (ITGB5) and integrin beta 8 (ITGB8). Both were expressed higher (15- and 6-fold, respectively) in glial precursors than in unpurified cells (the signal intensity of ITGB5 was 4712.6 in NAE and 301.7 in SM; the signal intensity of ITGB8 was 29.7 in NAE and 4.9 in SM).

### Transcription factors and molecules of major signal transduction pathways show distinct profile in A2B5+/NCAM- glial precursors

Screening of transcription factors, cell surface markers, receptors, and extracellular matrix molecules showed distinct profile for glial precursor population NAE. Sixty-one transcription factors were expressed at 2-fold or higher in glial precursors NAE, which included known transcription factors that drove glial specification such as Olig2, ID3 and Nkx2.2. GJA1 (Connexin 43), a known marker for glial precursors, was detected 4-fold higher in glial precursors (signal intensity 375.3) than in neuronal precursors (signal intensity 96.2).

We also performed a detailed pathway analysis by comparing the A2B5+/NCAM- glial precursors NAE to NSCs (WBFB, Table 6). Forty seven transcription factors were expressed higher in NAE than in WBFB. Among them were genes from the SLIT and NTRK-like family, SLITRK1, SLITRK4, and SLITRK5, which were expressed 5~10-fold higher in glial precursor NAE than in WBFB NSCs. In particular, expression signal value for SLITRK4 in NAE was 160 and was not detected in WBFB (signal intensity<0).

**Table 6:** Important pathways in NAE

	Symbol	NAE	WBFB	NAE/WBFB		Symbol	NAE	WBFB	NAE/WBFB
Transcription	SOX10	978.4	16.4	59.8	FGF	FGF12	116.3	8	14.5
factors	POU3F2	488.3	129.1	3.8		FGF9	58.2	16.9	3.4
					
	LZTS1	218.7	11.2	19.6	TGFβ	BAMBI	537.9	156.6	3.4
	DACH	175.9	0.3	703.4		CDKN2B	342.4	35	9.8
	SLITRK4	160.4	-1.2	∝		STAT1	274.1	124.7	2.2
	NR0B1	94.1	-8	∝		IGFBP3	159.4	22	7.3
	SLITRK5	91	8.9	10.3		COL1A2	127.9	46.7	2.7
	EMX1	82.7	12.7	6.5		BMP6	93.4	7.5	12.4
	SLIT2	51.4	23	2.2		TGFBI	49.6	4.1	12.2
					
Wnt	SFRP1	2241	638.9	3.5		SERPINE1	41.6	15.3	2.7
					
	SFRP2	259.5	51	5.1	cAMP	NPY	3923	167.9	23.4
	IL11	62.8	-2.3	∝		LDHA	3855	1923	2
					
Steroid	CRH	1990	41.2	48.4		SCG2	2126	137.9	15.4
related	HMGA1	381.8	157.5	2.4		SGK	595.2	250.9	2.4
	STAT1	274.1	124.7	2.2		PENK	433.9	-5.6	∝
	NR0B1	94.1	-8	∝		PLAT	367.9	155.8	2.4
					
MAPK	CDKN2B	342.4	35	9.8		CDKN2B	342.4	35	9.8
	CCNA1	334.5	44.5	7.5		CCNA1	334.5	44.5	7.5
	MAPK8IP2	162.8	81.1	2		BDNF	57.3	15.9	3.6
						CALB1	34.4	-4.1	∝

Two secreted frizzled-related proteins, SFRP1 and SFRP2, which are important antagonists of the Wnt pathway, were expressed three to five fold higher in the glial precursor NAE. On the contrary, a Wnt inducer, IL11, was also upregulated in NAE (signal intensity 62.8) and not detected in NSCs (WBFB, signal intensity <0). These data indicated that at least Wnt molecules were differentially regulated in NAE and direct evidence is needed to establish the role of SFRPs and other Wnt molecules in glial differentiation [30-32].

FGFs are important in both proliferation and differentiation of NSCs. Here we have found that FGF9 and FGF12 were highly expressed (3~14-fold) in glial precursors NAE compared to NSCs WBFB (signal intensity was 58.2 and 116.3 in NAE, and 16.9 and 8 in WBFB). Further examination of these two growth factors for their role in directing NSCs or glial precursors toward mature glia is warranted.

Steroids have been shown to affect and regulate glial gene expression [33]. We have found that corticotropin releasing hormone (CRH or CRF) was highly expressed in NAE (signal intensity 1989.8) but was 48-fold lower in WBFB (signal intensity 41.2). Additionally, expression of nuclear receptor family members such as NR0B1, was upregulated in NAE as compared to WBFB as well.

Several cell cycle regulators and key genes of the MAPK pathway, CDKN2B (cyclin-dependent kinase inhibitor 2B, or p15) which inhibits CDK4, and CCNA1 (Cyclin A1), were found to be expressed at 7–10 fold higher in NAE than in WBFB.

The TGFβ pathway is a key pathway involved in glial differentiation [16,34] and our findings were consistent with the published data. Expression of several key components of the TGFβ pathway, for example, nuclear receptor subfamily 0, group B, member 1 (NR0B1), bone morphogenetic protein 6 (BMP6), transforming growth factor beta-induced (TGFBI), and BMP and activin membrane-bound inhibitor homolog (BAMBI), was detected more than 10-fold higher in glial precursor NAE than in NSCs WBFB.

Cyclic AMP also plays an important role in neural and glial differentiation [35]. Several cAMP related genes, including calbindin 1 (CALB1), proenkephalin (PENK), neuropeptide Y (NPY) and secretogranin II (chromogranin C) (SCG2) were highly expressed in NAE but were expressed much lower (7~20-fold) or were absent in the NSC population WBFB.

## Discussion

In this manuscript, we generated global gene expression profiles for human glial precursors, astrocyte precursors, neuronal precursors and NSCs isolated from fetal tissues using Illumina bead array. We focused on detailed marker and pathway analysis for glial precursors by comparing their profile to other populations.

Differential expression of markers on the plasma membrane of glial precursors was used to isolate these cells from a general population of fetal brain cells. The general cellular population was depleted for those expressing NCAM followed by enrichment of those cells that were recognized by the A2B5 antibody. The resultant population (A2B5+ and NCAM-, NAE) has been shown to have characteristics of glial precursors and give rise to only oligodendrocytes and astrocytes upon further differentiation in vitro and in vivo (Figure 2 and data not shown).

Generally accepted glial precursor markers such as Olig1, Olig2, Nkx2.2, ID2, ID4, and glutamate transporter genes were expressed higher in the glial precursor populations (Table 4, see Additional file 1). Expression data collected for glial precursors, astrocyte precursors, neuronal precursors, and NSCs showed that glial precursors NAE possessed a distinct gene expression profile, suggesting several core signaling pathways that might be important in glial differentiation. For instance, major components of the TGFβ pathway, BMP6, BMP7, TGFBI, and TGFBII, were upregulated in glial precursors when compared to NSCs or neuronal precursors. These data are consistent with previous observations that BMPs can induce glial differentiation [16-18].

We also found several novel pathways or molecules that might be involved in glial differentiation. For instance, the SLIT and NTRK-like family proteins (Slitrk). Slitrk's are integral membrane proteins, with two leucine-rich repeat (LRR) domains similar to Slit family and C-term domains similar to Trk proteins and might possess the Trk kinase function. Genes of Slitrk family are found to be predominantly expressed in the brain and glial tumors including astrocytoma, oligodendroglioma and glioblastoma [36]. Consistent with these reports, three Slitrk proteins Slitrk1, Slitrk4 and Slitrk5, as well as Slit2 were differentially highly expressed in all glial precursor populations described in this work. These combined indicate that the Slit pathways are important to glial differentiation. Since Slitrk's are integral transmembrane proteins, these molecules may also serve as new cell surface markers for isolating glial precursors.

Similarly, Ephrins, proteins involved in axon guidance, were found to be differentially expressed in our glial precursor populations as well. For example, EPHA2, EPHA3, EPHA5, EPHB6 were expressed 2~4-fold higher in glial precursors than in NSCs or neuronal populations. In line with our data, the EphB2 receptor has recently been reported to be released at the neuron-glia interaction from neurons and endogenous EphB2 was expressed by glia simultaneously [37]. In addition, the Ephrin family receptor tyrosine kinases were shown to be upregulated in response to CNS injury as part of the function of glia [38]. The above mentioned pathways have previously been implicated in neuronal pathfinding and synaptogenesis. Their expression in glial lineages may reflect the growing body of evidence pointing to the importance of glia in neuronal development events [39,40].

Differential expression of genes in the Pax-Six-Eya-Dach (paired box-sine oculis homeobox-eyes absent-dachshund) gene regulatory network was also observed. Dach was expressed 700 fold higher in glial precursors than NSCs (Figure 4 and see Additional file 1), and Eya2 and Six5 were expressed 2~4-fold higher in glial precursors than in neuronal precursors (see Additional file 1). The Pax-Six-Eya-Dach network has been shown to be important for inner ear and other sensory organ development [41-44], it may be worthwhile to initiate studies that will elucidate this pathway's role in glial biology.

Several extracellular matrix metalloproteinases showed differential expression in glial populations and other cell types. For instance, MMP7 was expressed 40- to 200-fold higher in glial cells than in NSCs (Figure 4 and see Additional file 1). This is not totally unexpected as MMP7 has been reported to be involved in the canonical Wnt pathway as well as during FGF2-mediated response [45]. Expression of another metalloproteinase related molecule, tissue inhibitor of metalloproteinase 3 (TIMP3), was elevated in glial precursors as well (the signal intensity in NAE was 496.7). These data indicate that the metalloproteinase family may actively participate in glial differentiation.

Several frizzled-related protein molecules of the Wnt pathway, SFRP1, SFRP2 FRPB, IL11, and FRAT1, were highly expressed in glial precursors when compared to NSCs. However, similar expression was detected for these Wnt molecules in neuronal precursors, supporting the conclusion that these proteins are involved in general neural cell differentiation instead of glial specification.

Another gene that was brought to our attention was PENK (proenkephalin). It was expressed higher in glial precursors NAE and HOPC (signal intensities 400–3000) but was not detected in NSCs (signal intensity <0, Additional file 1). However, when we examined the expression levels of MAPK1 and MAPK3 (p38), major components of the MAPK-ERK pathway, which regulates PENK expression, no significant changes were found. Possible reasons might be that phosphorylation status or other posttranslational modifications are more important in determining the pathway activity than mRNA levels. To confirm this, proteomic analysis needs to be done as we and others have performed for several cell types including hESCs [46]. This is worth mentioning as protein expression and function are regulated at multiple levels while the present work focuses on the mRNA levels only.

Genes relevant to the signal transduction pathways Akt, CNTF, SDF-1/CXCR4, PDGF, glial cell line-derived nerve growth factor (GDNF) and receptor tyrosine kinase (RTK) were found to be differentially expressed as well. As expected, members of the PDGF pathway were expressed higher in glial precursors. For the EGF receptor family, NRG1 and EGFR, ERBB3, and the downstream effector SOS were also observed to be differentially upregulated in glial populations as compared to NSCs and neuronal precursors.

Recently, several zinc finger proteins have been implicated in various processes of neural development and glial differentiation [47,48]. Here we have found that zinc finger proteins Znf135, Znf136, Znf161, Znf24, Znf91, Znf435, Znf154, Znf232, and Znf219, were expressed as much as 14-fold or higher in glial precursors than in astrocyte precursors hAPC. Znf494, a zinc finger protein of unknown function [49] was expressed 415~663-fold higher in glial precursors than in NSCs. Conversely, another set of zinc finger proteins, Znf145, Znf537 Znf345, Znf370, Znf51 (Bcl6), Znf268, Znf375, and Znf373, were highly expressed in hAPC. The zinc fingers identified in this work may warrant further study.

Analyses of these expression data have led to the identification of novel cell surface markers which may be used for identification or isolation of glial precursors. Our results indicate that in addition to the well accepted surface marker NG2, Brevican and Integrin beta 5 and beta 8 are potential cell surface molecules for glial precursors. Brevican is a neural-specific chondroitin sulfate proteoglycan and is expressed in mature astrocytes and downregulated in adult rat spinal cords [50,51]. β integrins have been reported to be involved in spinal cord injury and regeneration process. Both Brevican and integrins can be regulated by the TGFβ pathway [50,52]. Our results show that Brevican expression is significantly increased in glial precursors. In line with this, expression of ADAMTS4, a gene that encodes a disintegrin and metalloproteinase that selectively cleaves Brevican/Lectican, also is elevated in the glial precursor population. A detailed time course study of its expression in glial development in the human fetal brain is underway to validate its utility as a marker for isolating glial precursors.

## Conclusion

We have compared gene expression profile of glial precursors to neuronal precursors, astrocyte precursors and NSCs. Such information may be utilized to further purify glial precursor populations, optimize media formulation, or study the effects of glial cell differentiation.

## Abbreviations

SM: staring material (unsorted fetal brain cells); NAE: NCAM flow through: A2B5 Eluate (A2B5+: PSA-NCAM- cells); AE: A2B5 Eluate (A2B5+ cells); NE: NCAM Eluate (PSA-NCAM+ cells); HOPC: human oligodendrocyte precursors (A2B5+: PSA-NCAM- cells); WB: Neurosphere from whole brain; FB: Neurosphere from frontal brain; hAPC: human astrocyte precursor cells (CD44+ cells); hESC: human embryonic stem cells; mESC: mouse embryonic stem cells; NSC: neural stem cells; FGF: fibroblast growth factor; EGF: epidermal growth factor; TGFβ: transforming growth factors beta; BMP: bone morphogenetic proteins; LIF: leukemia inhibitory factor; CNTF: ciliary neurotropic factor; Slitrk: SLIT and NTRK-like family proteins; PDGF: platelet derived growth factor; GDNF: glial cell line-derived nerve growth factor; RTK: receptor tyrosine kinase; MACS: magnetic cell sorting; FACS: fluorescence activate cell sorting;

## Authors' contributions

JTC and RWS provided and analyzed NAE, NE, AE, SM samples, participated in designing the project, drafting and editing the manuscript. WW provided and analyzed NAE, NE, AE, SM samples. HX provided hAPC sample and performed RT-PCR. JZ, FL, MZ performed bioinformatics study. JDC edited the manuscript. YL designed the project, coordinated the collaboration and drafted the manuscript. MSR conceived of the study and supervised YL. All authors read and approved the final manuscript.

## Supplementary Material

Additional file 1 **Gene expression of all samples included in this study.** Click here for file

Additional file 2 **Transcripts that are expressed at 2-fold or higher in NAE cells.** Click here for file
